# Testosterone Replacement Therapy in Women Is Associated with Improved Symptom Burden and Favorable Biomarker Changes: A Retrospective Observational Study

**DOI:** 10.3390/jpm16050231

**Published:** 2026-04-22

**Authors:** Carter W. Elggren, Charles H. Iverson, Madeline D. Morris, Ella F. Cooper-Leavitt, Genevieve Parker, Andrew W. Richardson, Asher P. Reynolds, Paul M. Cortes, Benjamin T. Bikman, Paul R. Reynolds

**Affiliations:** Department of Cell Biology and Physiology, Brigham Young University, Provo, UT 84602, USAecooperl@student.byu.edu (E.F.C.-L.);

**Keywords:** testosterone replacement therapy, women, biomarker, women’s health

## Abstract

**Background**: Testosterone is the most abundant biologically active sex steroid in women, yet the therapeutic implications of its age-related decline remain undercharacterized. Published trials have focused predominantly on sexual function, leaving gaps in understanding how testosterone replacement therapy (TRT) affects broader symptom domains and metabolic biomarkers in women. **Objective**: To investigate whether individualized, biomarker-guided TRT in women is associated with improvements across multiple symptom domains and favorable hormonal, hematologic, and cardiometabolic biomarker changes, and to examine whether symptomatic benefit varies with treatment duration. **Methods**: In this retrospective observational study, women (n = 332; ages 27 to 78; mean 45.7 ± 7.1 years) receiving TRT as part of routine clinical care through a telehealth-based platform completed a structured survey at a single post-treatment time point assessing eight symptom domains: energy/fatigue, memory, concentration, irritability, depression, anhedonia, sexual interest, and relationship satisfaction. Respondents were stratified by TRT duration (1 month to >12 months) and a subset (n = 120) underwent paired biomarker assessment at baseline and 12 weeks for total testosterone, free testosterone, SHBG, hemoglobin, and triglycerides. **Results**: Improvement was reported across all eight domains, with energy/fatigue showing the strongest response (84.3% improved). Depression, irritability, anhedonia, and sexual interest each exceeded 65% improvement. Cognitive domains showed a delayed trajectory, with meaningful gains emerging at 4 to 6 months. Quality of life improvement was reported by 89.7%, with significant improvement rising from 5.4% at 1 month to 51.5% at greater than 12 months. Energy/fatigue (64.2%) and mood (49.7%) ranked above sexual desire (41.3%) as self-identified areas of greatest benefit. All five biomarkers changed favorably: total testosterone +151.8% (d = 3.60), free testosterone +216.7% (d = 3.01), hemoglobin +5.5% (d = 2.03), SHBG −13.3% (d = 1.57), and triglycerides −12.6% (d = 1.28). **Conclusions**: Individualized TRT in women was associated with broad symptomatic improvement spanning energy/fatigue, depression, irritability, anhedonia, cognitive function, and sexual interest, with duration-dependent gains and favorable biomarker changes across all five markers assessed. These findings suggest that the value of testosterone in women extends beyond sexual function and supports the need for larger controlled trials with extended follow-up.

## 1. Introduction

Testosterone is the most abundant biologically active sex steroid in women throughout the female lifespan, with circulating concentrations approximately 10-fold higher than estradiol even during peak reproductive years [1,2]. In premenopausal women, testosterone is produced by the ovaries and adrenal glands at a rate of approximately 300 µg per day, and it exerts broad physiological effects on skeletal muscle, bone mineral density, central nervous system function, lipid metabolism, erythropoiesis, and sexual health [3,4]. Despite this foundational role, testosterone has historically received far less clinical and investigative attention in women than in men, leaving substantial gaps in our understanding of its decline, the consequences of that decline, and the therapeutic potential of its restoration.

Serum testosterone concentrations in women decrease progressively with age, beginning in the third decade and continuing through the menopausal transition and beyond [5,6]. Davison et al. demonstrated that women aged 45 and older exhibit total testosterone levels approximately 50% lower than women in their twenties, with corresponding reductions in bioavailable fractions [5]. Surgical menopause (bilateral oophorectomy) produces an even more abrupt reduction, as the ovarian contribution to testosterone production is eliminated entirely [7]. This decline is not merely biochemical. Testosterone insufficiency in women has been associated with a constellation of symptoms including diminished energy and vitality, reduced sexual desire and arousal, depressed mood, impaired concentration and memory, loss of lean body mass, and increased central adiposity [3,8,9]. These symptoms overlap considerably with those attributed to estrogen deficiency during menopause, and there is growing recognition that testosterone loss may be an underappreciated contributor to the symptom burden experienced by peri- and postmenopausal women [4,10].

From a metabolic standpoint, testosterone plays a modulatory role in insulin sensitivity, lipid homeostasis, and inflammatory signaling. Observational studies have shown that higher endogenous testosterone concentrations in postmenopausal women are associated with more favorable lipid profiles, including lower triglycerides and higher HDL cholesterol [11,12]. The relationship between testosterone and systemic inflammation remains complex and dose-dependent; however, at physiologic concentrations, androgens appear to exert anti-inflammatory effects through suppression of pro-inflammatory cytokines and modulation of adipocytokine signaling [13,14]. These metabolic and inflammatory pathways are particularly relevant in midlife women, for whom chronic low-grade inflammation, or “inflammaging,” represents a convergent risk factor for cardiovascular disease, metabolic syndrome, and cognitive decline [15,16].

It is important to distinguish the physiologic restoration of testosterone pursued in TRT from the pathophysiologic androgen excess characteristic of conditions such as polycystic ovary syndrome (PCOS). In PCOS, chronically elevated endogenous testosterone—arising in the context of insulin resistance, dysregulated gonadotropin signaling, and altered steroidogenesis—is associated with dyslipidemia, hypertension, central obesity, and increased cardiovascular risk [17,18]. Experimental models further support this relationship, as PCOS-like phenotypes can be induced through exogenous androgen administration or aromatase inhibition, resulting in elevated endogenous testosterone and adverse metabolic sequelae [19,20]. The cardiometabolic consequences of PCOS-associated hyperandrogenism therefore reflect a fundamentally different hormonal milieu than the targeted, biomarker-guided restoration of testosterone to premenopausal physiologic concentrations pursued in the present study. The therapeutic objective here is not androgen excess but correction of androgen insufficiency, and the targeted dose range, 25 to 50 ng/dL total testosterone, approximates the upper physiologic range for premenopausal women rather than the supraphysiologic concentrations associated with adverse outcomes. This dose–context distinction is central to interpreting the metabolic findings reported below. The clinical evidence base for TRT in women, while growing, remains limited relative to the male literature [3,14,21]. The landmark Global Consensus Position Statement published in 2019 acknowledged a role for testosterone therapy in postmenopausal women with hypoactive sexual desire disorder (HSDD), recommending transdermal formulations that approximate premenopausal physiologic concentrations [21]. The International Society for the Study of Women’s Sexual Health (ISSWSH) subsequently issued clinical practice guidelines reinforcing this recommendation and providing a monitoring framework centered on serum total testosterone, free testosterone or free androgen index (FAI), and safety markers including lipid panels and complete blood counts [22]. A comprehensive meta-analysis of 36 randomized controlled trials (RCTs) encompassing 8480 women confirmed that testosterone therapy was associated with meaningful improvements in sexual function, including desire, arousal, orgasm, and satisfaction, while also identifying reductions in total cholesterol and triglycerides [12]. These findings notwithstanding, the majority of published RCTs have focused narrowly on sexual function endpoints, and relatively few have examined the broader symptomatic and metabolic effects of TRT in women across multiple domains simultaneously. The present study was designed to address these gaps. The aim was to characterize multi-domain symptomatic outcomes across varying TRT durations and to assess hormonal, hematologic, and cardiometabolic biomarker changes at 12 weeks in a subset of participants receiving individualized TRT through a telehealth-based platform as part of routine clinical care. These findings contribute real-world evidence to support personalized, biomarker-driven approaches to hormone optimization in women and identify priorities for future controlled trials.

## 2. Materials and Methods

### 2.1. Study Design

This was a retrospective observational study of adult women receiving individualized TRT as part of routine clinical care, conducted through a telehealth-based platform affiliated with Joi + Blokes (Nashville, TN, USA), a direct-to-consumer telehealth company specializing in biomarker-guided hormone optimization and personalized wellness care for both women and men. The 12-week biomarker interval was selected based on prior evidence demonstrating detectable hormonal, hematologic, and lipid changes within 8 to 12 weeks of testosterone initiation in women [7,12]. All clinical protocols, including blood testing, testosterone administration, and symptom monitoring, represented standard care and were not research-imposed.

### 2.2. Participants

Eligible participants were adult women who had initiated TRT through the telehealth platform and completed the symptom survey as part of routine clinical monitoring. Inclusion required: (1) female sex; (2) age 18 years or older; and (3) active enrollment in the platform’s TRT program at the time of survey administration. Exclusion criteria included acute illness at the time of baseline biomarker assessment, recent history of active infection, immunosuppressive therapy, or major medical intervention within 30 days of enrollment, and current pharmaceutical treatment for active autoimmune or inflammatory conditions. No exclusions were made based on the presence of diabetes mellitus, metabolic syndrome, or obesity, reflecting the real-world clinical population served by the platform. Participants were not excluded based on menopausal status, age, or route of testosterone administration. All data were obtained as part of routine care, and the retrospective analysis was conducted using de-identified records without additional patient contact.

A total of 332 women met eligibility criteria and completed the symptom survey (age range 27 to 78 years; mean 45.7 ± 7.1; median 45). The cohort was stratified by TRT duration: 1 month (n = 37), 1 to 3 months (n = 112), 4 to 6 months (n = 80), 7 to 12 months (n = 70), and greater than 12 months (n = 33). Age distribution: less than 40 years, 55 (16.6%); 40 to 49 years, 166 (50.0%); 50 to 59 years, 88 (26.5%); 60 years and older, 23 (6.9%). A subset of 120 participants from the 1 to 3 month duration group additionally had complete paired laboratory data available at both baseline and 12 weeks as part of routine clinical monitoring and were therefore included in the biomarker analysis. This subset was identified retrospectively based on data availability and was not defined by pre-specified selection criteria beyond those applied to the full cohort. Participants in other duration groups lacked complete paired baseline and 12-week biomarker data within the dataset and were therefore not included in the biomarker analysis. The biomarker subset (n = 120) was drawn exclusively from the 1 to 3 month duration group (n = 112 in the symptom cohort), representing those early-treatment participants for whom complete laboratory records happened to be available. No formal comparison of baseline characteristics between the biomarker subset and the full symptom cohort was performed, as individual-level demographic and clinical covariate data were not available. The extent to which this subset is representative of the broader cohort therefore cannot be confirmed, and findings from the biomarker analysis should be interpreted with this uncertainty in mind.

### 2.3. Clinical Exposure

All participants received individualized TRT as part of routine clinical care through the telehealth platform, with the objective of restoring serum testosterone to physiologic premenopausal concentrations while alleviating symptoms of testosterone insufficiency. Prior to initiating TRT, all participants underwent baseline laboratory assessment including serum total testosterone, free testosterone, SHBG, complete blood count, and lipid panel. Clinical eligibility for TRT was determined by a licensed provider based on the combination of symptom burden, baseline hormone levels, and individual clinical history. Testosterone was administered via one of three modalities selected based on patient preference and provider clinical judgment: (1) topical preparations, including compounded testosterone cream or gel (typically 1–10 mg/day), applied daily to skin; (2) oral formulations including compounded testosterone troches, orally disintegrating tablets, or rapidly dissolving tablets (typically 0.5–2 mg per dose), administered once or twice daily; or (3) subcutaneous injections of compounded testosterone cypionate or enanthate in oil (typically 2–10 mg per injection), administered once or twice weekly. All formulations were sourced through licensed compounding pharmacies operating under applicable state and federal regulations and prescribed by licensed providers through the telehealth platform. Starting doses were determined by the prescribing provider based on baseline testosterone levels and clinical presentation. Dose titration targeted serum total testosterone concentrations of 25 to 50 ng/dL [5,21,22], representing the upper physiologic range for premenopausal women. Follow-up laboratory assessments were performed at approximately 12 weeks and at subsequent quarterly or monthly intervals, with dose adjustments made by the provider based on laboratory results, clinical response, and side effect profile. Delivery modality distribution across the cohort was not captured in the survey instrument and therefore cannot be reported. Participants may have received concurrent micronutrient supplementation (e.g., vitamin D, omega-3 fatty acids, B vitamins) guided by baseline biomarker profiles, consistent with the clinical model described previously [23]. However, testosterone replacement was the primary and unifying clinical exposure of interest, and the present analysis focuses specifically on outcomes associated with TRT.

### 2.4. Symptom Assessment

Subjective outcomes were assessed via a structured survey evaluating eight domains: energy/fatigue, memory, concentration, irritability, depression, anhedonia, sexual interest, and relationship satisfaction. The survey was self-administered electronically through the telehealth platform, completed by participants independently without clinician assistance at the time of a routine clinical monitoring contact.

Each domain was rated on a 6-point scale (0 = not at all, 1 = much worse, 2 = slightly worse, 3 = no change, 4 = somewhat improved, 5 = significantly improved) and categorized as Improved (4 or 5), No Change (3), or Worsened (0, 1, or 2). Responses reflect each participant’s retrospective self-perception of change since initiating TRT rather than objectively measured pre-to-post differences, as no baseline symptom assessment was administered. Participants also rated overall quality of life (QoL) change on a 5-point scale (significantly improved to worsened) and identified up to three areas of greatest improvement from a predefined list. The survey was administered at a single time point based on current treatment status. Because symptoms were assessed at a single post-treatment time point without individual baseline measurement, stratification by TRT duration enables examination of temporal patterns across the cohort but does not constitute longitudinal follow-up of individual participants and cannot capture within-individual change from pre-treatment status.

### 2.5. Biomarker Assessment

Blood samples were collected at two time points in a subset of 120 participants: at baseline, prior to TRT initiation, and at 12 weeks following TRT initiation, coinciding with the first scheduled follow-up laboratory assessment per the platform’s standard clinical monitoring protocol.

Sample Collection: Serum samples for hormonal and metabolic analyses were collected in serum separator tubes (SST), allowed to clot for 30 to 60 min at room temperature, centrifuged at 1000 to 2000× *g*, and refrigerated at 2 to 8 °C until analysis. Whole blood for hematologic analysis was collected in EDTA tubes and transported for analysis within 24 to 48 h of collection. All collection and handling procedures followed CLIA guidelines.

Biochemical Analysis: Samples were analyzed at CLIA-certified reference laboratories (LabCorp, Quest Diagnostics, Bioreference Health; Burlington, NC, USA) using standardized clinical assays. Total testosterone was measured by liquid chromatography-tandem mass spectrometry (LC-MS/MS), consistent with Endocrine Society recommendations for accuracy in female reference ranges. Free testosterone was calculated from total testosterone and SHBG using the Vermeulen equation. SHBG was measured by immunochemiluminometric assay. Hemoglobin was measured as part of a standard complete blood count using automated hematology analyzers. Triglycerides were measured by enzymatic colorimetric assay as part of a standard lipid panel. Because analyses were performed as part of routine clinical care across three independent reference laboratories, the specific instrument models, reagent lots, and manufacturers used for each assay were determined by the individual laboratory and were not uniformly recorded or available to the research team. All laboratories operated under CLIA certification, ensuring adherence to standardized quality control and proficiency testing requirements.

Five biomarkers were selected based on physiological responsiveness to testosterone and clinical relevance in women: (1) serum total testosterone, as the primary indicator of dose adequacy; (2) free testosterone, reflecting bioavailable androgen activity [7,22]; (3) sex hormone-binding globulin (SHBG), as a modulator of testosterone bioavailability and predictor of treatment response [21,22]; (4) hemoglobin, as a downstream marker of testosterone-stimulated erythropoiesis [24]; and (5) triglycerides, as a marker of cardiometabolic risk previously shown to improve with testosterone therapy in women [12]. Complete blood counts and full lipid panels were additionally monitored per standard clinical care, reflecting both their physiological relevance as downstream markers of testosterone action, given testosterone’s known erythropoietic effects and influence on lipid metabolism, and their utility in clinical surveillance for hematologic and cardiometabolic changes during TRT.

### 2.6. Data Analysis

Symptom data were analyzed using descriptive statistics including means, standard deviations, and proportional response distributions, stratified by TRT duration. Because no baseline symptom assessment was performed, these data represent retrospective perceptions of change and do not constitute measured within-individual differences over time. No formal a priori sample size calculation was performed, as the study population was determined by the availability of de-identified clinical records meeting eligibility criteria within the dataset rather than by prospective power analysis. The sample sizes reported reflect the totality of eligible participants available within the specified duration strata. For the biomarker subset (n = 120), normality of all continuous variables was assessed prior to analysis using the Shapiro–Wilk test. All five biomarker variables—total testosterone, free testosterone, SHBG, hemoglobin, and triglycerides—satisfied the assumption of normality at both baseline and 12 weeks (all *p* > 0.05), supporting the use of paired two-tailed *t*-tests for within-group comparisons. Effect sizes were calculated as Cohen’s d for paired samples, and 95% confidence intervals were computed for mean changes. Given the exploratory nature and limited number of comparisons (five paired tests), no adjustment for multiple comparisons was applied. Individual-level covariate data—including BMI, menopausal status, comorbidities, and concurrent medications—were not available in the dataset and therefore adjusted regression modeling could not be performed. This represents a meaningful analytic limitation; future studies should incorporate covariate-adjusted models to better account for potential confounding. The duration-response analysis employed cross-sectional stratification by self-reported TRT duration. This design permits examination of temporal patterns but does not establish within-individual longitudinal trajectories; potential confounding by indication (participants experiencing greater benefit may be more likely to continue therapy) is acknowledged. Statistical analyses were performed with GraphPad Prism 8.0 software.

### 2.7. Ethical Considerations

This study was conducted as part of a clinical quality improvement initiative within routine care operations and did not involve experimental pharmaceuticals or research-assigned procedures. The symptom survey was administered to patients as part of standard clinical monitoring by the telehealth platform and was not deployed as a research instrument. The retrospective analysis was subsequently conducted on de-identified records without additional direct patient contact, qualifying for IRB exemption under 45 CFR 46.104(d)(2), which covers research involving survey procedures in which the only involvement of human subjects is through the use of surveys or interviews and the information obtained is recorded in a manner such that participants cannot be identified.

Written informed consent was not obtained for the following reasons: (1) the survey was administered within the context of routine clinical care, not as part of a prospectively designed research study; (2) blood samples were collected solely as part of standard clinical monitoring and were not obtained for research purposes; (3) the retrospective analysis used fully de-identified data; and (4) the study met criteria for IRB exemption under U.S. federal regulations (45 CFR 46.104(d)(2) and (d)(4)), which explicitly do not require informed consent when research involves only the retrospective analysis of de-identified records generated during routine care and identifiability risk has been eliminated through de-identification. This regulatory framework is widely applied in retrospective clinical research and does not constitute a deviation from standard ethical norms.

De-identification was performed in accordance with HIPAA Safe Harbor standards (45 CFR §164.514(b)), which required the removal of all 18 categories of protected health information prior to analysis. These included: names, geographic identifiers smaller than state, dates directly related to individuals (including birth dates and dates of service), telephone and fax numbers, email addresses, Social Security numbers, medical record and account numbers, health plan beneficiary numbers, certificate and license numbers, vehicle identifiers, device identifiers and serial numbers, URLs, IP addresses, biometric identifiers, full-face photographs, and any other unique identifying numbers or codes. Following de-identification, no member of the research team had access to a linkage key or re-identification mechanism. All data used in the preparation of this manuscript were in fully de-identified form throughout the analysis.

## 3. Results

### 3.1. Participant Characteristics

A total of 332 women completed the symptom survey. Demographic and clinical characteristics are summarized in Table 1. The mean age was 45.7 ± 7.1 years (median 45; range 27 to 78), with half of participants (50.0%) in the 40 to 49 year age group. The largest TRT duration subgroup was 1 to 3 months (33.7%). A subset of 120 participants underwent paired biomarker assessment at baseline and 12 weeks.

### 3.2. Symptom Outcomes

**Figure 1:**
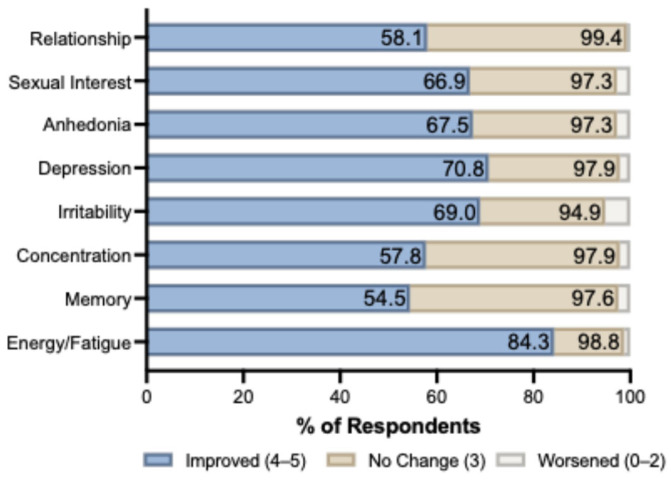
Symptom response distribution across eight domains in women receiving testosterone replacement therapy (n = 332). Participants rated change in each domain on a 6-point scale: 0 = not at all, 1 = much worse, 2 = slightly worse, 3 = no change, 4 = somewhat improved, 5 = significantly improved. Responses were categorized as Improved (scores 4 or 5; blue), No Change (score 3; tan), or Worsened (scores 0, 1, or 2; white). Numbers within bars indicate the percentage of respondents in each category. Energy/fatigue showed the highest improvement rate (84.3%), while memory and concentration showed the highest proportions of no change (43.1% and 40.1%, respectively), consistent with a slower onset of cognitive effects.

Participants reported improvement across all eight symptom domains (Figure 1). Energy/fatigue showed the strongest response, with 84.3% reporting improvement and a mean score of 4.17 ± 0.73. Depression (70.8%; mean 3.99 ± 0.87), irritability (69.0%; mean 3.91 ± 0.87), anhedonia (67.5%; mean 3.90 ± 0.90), and sexual interest (66.9%; mean 3.87 ± 0.84) all exceeded 65% improvement. Cognitive domains showed comparatively lower rates: concentration 57.8% (mean 3.70 ± 0.76) and memory 54.5% (mean 3.63 ± 0.74). Relationship satisfaction was improved in 58.1% (mean 3.80 ± 0.79). Worsening was rare across all domains, ranging from 0.6% (relationship satisfaction) to 5.1% (irritability). More than 94% of respondents reported either improvement or no change in every domain assessed.

### 3.3. Duration-Response Relationships

**Figure 2:**
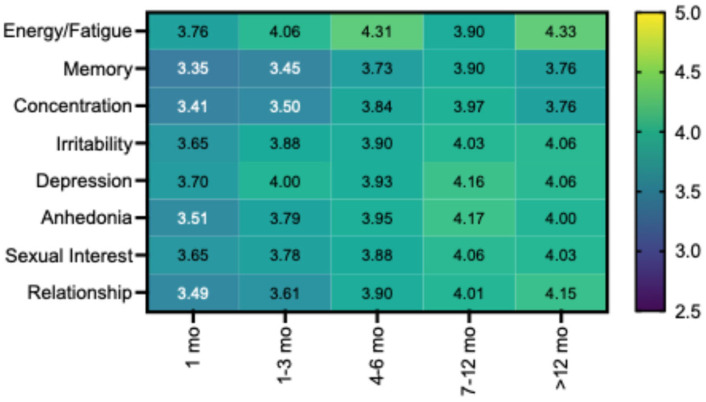
Mean symptom scores by TRT duration across eight domains. Scores range from 0 (not at all/much worse) to 5 (significantly improved), with 3 representing no change. Participants were stratified into five duration groups: 1 month (n = 37), 1–3 months (n = 112), 4–6 months (n = 80), 7–12 months (n = 70), and >12 (12–18) months (n = 33). Data points represent group means. Energy/fatigue demonstrated the earliest and most robust response, achieving near-maximal improvement by 4–6 months. Cognitive domains (memory, concentration) exhibited a delayed response trajectory, with meaningful gains emerging at 4–6 months and continuing through 7–12 months. The modest attenuation observed in several domains beyond 12 months may reflect selection effects, symptom adaptation, or smaller sample size in the longest-duration group.

Stratification by TRT duration revealed progressive improvement across all eight domains (Figure 2). Energy/fatigue showed the earliest and most robust trajectory, rising from 3.76 at 1 month to 4.31 at 4 to 6 months and plateauing thereafter. Mood-related domains (depression, irritability, anhedonia) demonstrated steady gains through 7 to 12 months. Sexual interest and relationship satisfaction followed similar upward trajectories across the treatment timeline. Cognitive domains exhibited a notably delayed response. Memory and concentration started at the lowest scores of any domain at 1 month (3.35 and 3.41, respectively), with meaningful gains emerging at 4 to 6 months and continuing through 7 to 12 months. This delayed inflection point suggests that the neurological mechanisms underlying testosterone’s cognitive effects may require longer treatment duration to manifest relative to energy, mood, and sexual function. The proportion reporting improvement also increased with duration. For energy/fatigue, the rate rose from 67.6% at 1 month to 90.9% at greater than 12 months. Memory increased from 35.1% to 60.6%, concentration from 37.8% to 69.7%, and sexual interest from 56.8% to 78.8%. A modest softening in several domains beyond 12 months, most evident in memory and concentration, may reflect selection effects, smaller sample size in the longest duration group (n = 33), or a true plateau in benefit.

### 3.4. Quality of Life (QoL)

**Figure 3:**
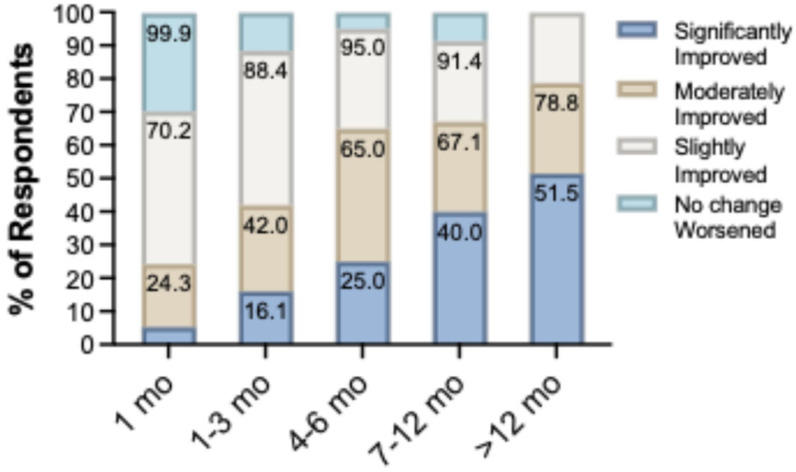
Quality of life (QoL) improvement by TRT duration. Participants rated overall change in QoL on a 5-point scale: significantly improved, moderately improved, slightly improved, no change, or worsened. Stacked bars represent the percentage of respondents in each category within each duration group. Values at the top of bars indicate the cumulative percentage reporting any degree of improvement. The proportion reporting significant improvement increased progressively from 5.4% at 1 month to 51.5% at >12 (12—18) months, while the combined rate of any improvement rose from 70.2% to 100%. Only one participant across the entire cohort (0.3%) reported worsened QoL.

Overall QoL improvement was reported by 89.7% of participants: 25.6% significantly improved, 28.9% moderately improved, and 35.2% slightly improved. Only 9.9% reported no change, and a single participant (0.3%) reported worsening. A pronounced dose–response relationship emerged when stratified by TRT duration (Figure 3). At 1 month, 70.2% reported any improvement but only 5.4% characterized it as significant. By greater than 12 months, 51.5% reported significant improvement, with no participants reporting no change or worsening. The progressive shift from “slightly improved” toward “significantly improved” indicates that while initial QoL gains occur early, the perceived magnitude of benefit continues to accrue with sustained therapy. Importantly, the 48.5% of participants in the greater than 12 month group who did not report significant QoL improvement should not be interpreted as having experienced no benefit; at this duration, no participant reported no change or worsening, meaning the entirety of this subgroup reported at least moderate or slight improvement. These gradations of self-reported QoL improvement likely reflect clinically meaningful differences in symptom burden reduction, consistent with the domain-specific score trajectories observed across the cohort.

### 3.5. Self-Identified Improvement Areas

**Figure 4:**
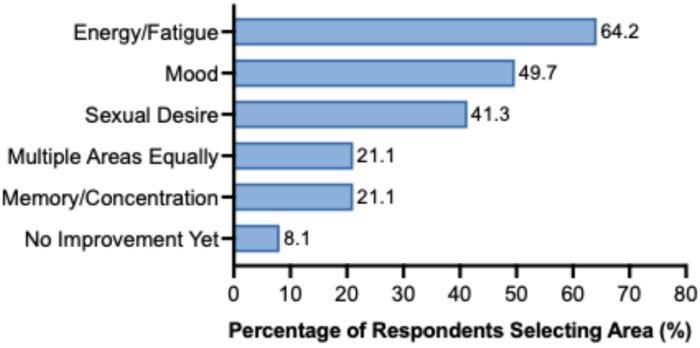
Self-identified top improvement areas among women receiving testosterone replacement therapy (n = 332). Participants selected up to three areas in which they experienced the greatest benefit. Energy/fatigue was the most frequently selected area (64.2%), followed by mood (49.7%) and sexual desire (41.3%). Memory/concentration and multiple areas equally were each selected by 21.1% of respondents. A total of 8.1% reported no improvement at the time of survey completion.

Participants most frequently identified energy/fatigue (64.2%) as their area of greatest improvement, followed by mood (49.7%) and sexual desire (41.3%) (Figure 4). Memory/concentration was selected by 21.1%, and 8.1% reported no improvement at the time of survey completion.

### 3.6. Biomarker Analyses

Paired biomarker data from the 120-participant subset are presented in Table 2. All five markers changed in the expected physiological direction over 12 weeks. Total testosterone increased from 16.8 ± 4.4 to 42.3 ± 8.7 ng/dL (+151.8%; d = 3.60), and free testosterone from 1.2 ± 0.3 to 3.8 ± 0.9 pg/mL (+216.7%; d = 3.01), confirming pharmacological activity. SHBG decreased by 13.3% (d = 1.57), indicating improved androgen bioavailability beyond what the total testosterone increase alone would suggest. Hemoglobin increased from 12.7 ± 0.6 to 13.4 ± 0.7 g/dL (+5.5%; d = 2.03). All post-treatment values remained within normal limits. Triglycerides decreased from 159.6 ± 30.5 to 139.5 ± 34.5 mg/dL (−12.6%; d = 1.28). Responder analysis demonstrated 100% improvement in total and free testosterone. For downstream markers, 86.7% showed meaningful hemoglobin increase, 77.5% meaningful SHBG reduction, and 74.2% meaningful triglyceride reduction. Worsening was observed in only 1 participant for SHBG and 4 for triglycerides.

## 4. Discussion

This study presents multi-domain symptomatic and biomarker outcomes from 332 women receiving individualized TRT in a real-world clinical setting. The findings suggest that TRT is associated with broad symptomatic improvement extending well beyond sexual function, with a clear duration-response relationship and favorable metabolic and hematologic biomarker changes at 12 weeks. These results add to growing evidence supporting the therapeutic potential of testosterone in women while highlighting domains underexplored in the existing literature.

### 4.1. Symptomatic Outcomes

Energy/fatigue was the most responsive domain, with 84.3% reporting improvement and the highest mean score of any domain assessed. This finding is consistent with earlier RCTs: Goldstat et al. reported significant improvements in well-being and mood in premenopausal women receiving transdermal testosterone [8], and Davis and Wahlin-Jacobsen identified fatigue and diminished vitality as core features of testosterone insufficiency distinct from estrogen-related symptoms [3]. The strength of the energy response, and its ranking as the most frequently self-identified area of improvement (64.2%), suggests that fatigue may be an underappreciated primary indication for TRT in women.

Depression, irritability, and anhedonia all exceeded 65% improvement, consistent with evidence for androgen receptor-mediated effects on mood regulation. Testosterone modulates serotonergic and dopaminergic neurotransmission, and androgen receptors are expressed throughout limbic structures involved in emotional processing [25,26]. The ADORE trial demonstrated that transdermal testosterone significantly improved mood and reduced depressive symptoms in postmenopausal women independently of sexual function effects [27]. Our findings align with these observations and extend them across a broader age range and multiple treatment durations.

The delayed cognitive response pattern is among the more novel findings of this study. Memory and concentration started at the lowest baseline scores and did not show meaningful gains until 4 to 6 months. This is biologically plausible as testosterone influences hippocampal neurogenesis, synaptic plasticity, and BDNF expression through both androgen receptor activation and local aromatization to estradiol [28,29], processes that operate on a slower timescale than the receptor-mediated effects likely driving earlier energy and mood improvements. Cherrier et al. demonstrated that exogenous testosterone improved verbal memory and spatial performance in older adults with cognitive impairment over weeks to months [30]. Our cross-sectional data are consistent with this timeline, though longitudinal confirmation is needed.

Sexual interest improved in 66.9% of participants, aligning with the established evidence base. The Islam et al. Lancet meta-analysis of 36 RCTs in 8480 women confirmed significant improvements in desire, arousal, orgasm, and satisfaction [12], forming the basis for the ISSWSH clinical practice guidelines recommending testosterone for postmenopausal HSDD [22]. Our data are consistent with these findings. However, participants ranked energy/fatigue and mood above sexual desire as their primary areas of improvement, suggesting that the current clinical and regulatory framing of TRT in women may underrepresent the breadth of its therapeutic value. Notably, 8.1% of participants reported no improvement at the time of survey completion. This proportion, while modest, is clinically meaningful and likely reflects genuine heterogeneity in treatment response. Potential explanations include interindividual variation in androgen receptor sensitivity, differences in baseline hormonal milieu, comorbid conditions not captured in the dataset, suboptimal dose titration at the time of survey, or insufficient treatment duration in some participants. This underscores the importance of thorough baseline evaluation of clinical and metabolic risk factors prior to TRT initiation, and suggests that future studies should prospectively characterize predictors of non-response to better identify candidates most likely to benefit from individualized testosterone therapy.

### 4.2. Biomarker Outcomes

The hormonal markers confirm pharmacologic efficacy. The mean post-treatment total testosterone of 42.3 ng/dL falls within the physiologic premenopausal range described by Davison et al. [5], and the free testosterone increase from 1.2 to 3.8 pg/mL is comparable to the shift observed by Shifren et al. in oophorectomized women receiving transdermal testosterone (1.2 ± 0.8 to 3.9 ± 2.4 pg/mL) [7]. The concurrent 13.3% SHBG reduction further augmented androgen bioavailability, a clinically relevant effect given that elevated SHBG predicts non-response to testosterone therapy in women [21,22].

The hemoglobin increase of 0.67 g/dL reflects testosterone’s erythropoietic effect, mediated through renal EPO upregulation and direct stimulation of erythroid progenitor cells [24,31]. Bhasin et al. established a clear dose–response relationship between testosterone and erythropoiesis [32], and current guidelines recommend CBC monitoring specifically for this reason [22]. The magnitude observed here is proportionally smaller than those reported at supraphysiologic doses [24,31], and all post-treatment values remained within normal limits. Even modest improvements in oxygen-carrying capacity may contribute meaningfully to the energy improvements reported by participants, particularly in women with baseline hemoglobin in the low-normal range.

The triglyceride reduction of 12.6% is consistent with published RCT data. The Islam et al. meta-analysis identified significant triglyceride reductions with testosterone therapy in women [12], and Davis et al. found an inverse association between endogenous testosterone and triglyceride levels in 3231 older women in the SHOW study [11]. The mechanism likely involves testosterone’s effects on hepatic lipase activity and visceral adipose metabolism [33]. This carries clinical significance given that hypertriglyceridemia is an independent cardiovascular risk factor in women and that triglyceride levels tend to rise through the menopausal transition [34]. These favorable cardiometabolic findings should be interpreted in the context of the broader literature on androgens and metabolic health in women, which at first glance may appear contradictory. Hyperandrogenic conditions such as PCOS are well-established as conferring adverse cardiometabolic risk, including dyslipidemia, insulin resistance, and elevated cardiovascular risk [17,18]. However, the metabolic consequences of PCOS-associated androgen excess arise within a pathophysiologic hormonal environment characterized by insulin resistance, dysregulated gonadotropin signaling, and chronically supraphysiologic androgen concentrations—a context fundamentally distinct from the physiologic restoration pursued here. The dose-dependent and context-dependent nature of testosterone’s metabolic effects in women is well recognized; at physiologic concentrations achieved through biomarker-guided titration, androgens appear to exert favorable effects on lipid metabolism and insulin sensitivity, whereas pathophysiologic excess produces the opposite [35,36]. The present findings are consistent with this dose-context framework and should not be conflated with the metabolic consequences of hyperandrogenism.

### 4.3. Duration-Response Implications

The duration-response patterns have practical clinical implications. The progression of significant QoL improvement from 5.4% at 1 month to 51.5% at greater than 12 months argues against premature discontinuation based on early assessment, particularly for cognitive outcomes where meaningful gains did not emerge until 4 to 6 months. Many published RCTs have used treatment durations of 12 to 24 weeks, which may be insufficient to capture the full benefit spectrum. Future trial designs should consider extended follow-up of at least 6 to 12 months to characterize the temporal dynamics of TRT response in women.

### 4.4. The Role of Personalized, Biomarker-Guided Hormone Optimization

A distinguishing feature of this study is the personalized, biomarker-driven framework within which TRT was administered. Unlike fixed-dose RCT designs, the protocol individualized dosing to each participant’s baseline hormone levels, symptom profile, and clinical response, with iterative titration guided by serial laboratory monitoring. This reflects an emerging model of precision hormone optimization in which real-time biological data inform treatment decisions rather than relying on population-level dosing standards.

The rationale is grounded in the considerable interindividual variability in testosterone metabolism, SHBG binding, receptor sensitivity, and downstream tissue response across women [3,21]. A fixed dose that achieves therapeutic concentrations in one patient may be subtherapeutic or excessive in another. By anchoring decisions to objective biomarker feedback, the personalized model aims to optimize the benefit-to-risk ratio for each individual, paralleling emerging trends in precision nutrition and biomarker-guided supplementation [23,37].

The broad improvement observed across energy, mood, cognition, sexual health, and metabolic biomarkers is consistent with the hypothesis that individualized protocols may capture a wider range of benefit than standardized regimens. Concurrent safety monitoring ensures that favorable changes do not come at the expense of adverse effects. These findings, together with our previous report on personalized supplementation and inflammatory biomarker reduction [23], support a broader paradigm in which biomarker-guided, individualized interventions may offer meaningful advantages over one-size-fits-all approaches to health optimization in midlife and older adults.

### 4.5. Limitations

Several limitations must be acknowledged, and their collective impact on the interpretability of findings should be weighted carefully rather than treated as minor caveats.

With respect to study population and selection, participants were drawn from a single telehealth-based platform serving women who had already initiated and remained in TRT-based clinical care. This introduces selection bias at the level of enrollment: the cohort does not represent all women who initiated TRT, and those who discontinued—whether due to lack of benefit, adverse effects, or other reasons—are systematically absent. The cross-sectional stratification by TRT duration compounds this issue, as longer-duration groups are necessarily composed of individuals who chose to continue therapy. For the symptom cohort, women who discontinued TRT prior to survey administration, whether due to side effects, lack of perceived benefit, cost, or other factors, are entirely absent from the dataset. As a result, each duration stratum captures only those women who were still actively engaged in care at that time point, and longer-duration groups are, by definition, composed of individuals who opted to continue therapy. In our data, the progressive improvement in symptom scores and quality of life observed across duration groups may therefore reflect this accumulation of favorable responders rather than a true treatment effect that accrues over time. For the biomarker subset specifically, the restriction to participants with complete paired laboratory records introduces an additional layer of selection: women who did not return for 12-week follow-up testing are excluded, which may further skew the biomarker findings toward a more favorable profile.

Regarding outcome measurement, symptom outcomes were assessed at a single post-treatment time point using a non-validated, study-specific survey instrument. Responses reflect retrospective self-perceptions of change since initiating TRT rather than objectively measured pre-to-post differences. The absence of a baseline symptom assessment means that true within-individual change cannot be established and that responses are subject to recall bias, social desirability effects, and the general tendency to perceive improvement in the context of an actively pursued treatment. The survey instrument has not been validated against established psychometric tools such as the PROMIS fatigue scale, the PHQ-9, or the Female Sexual Function Index, limiting comparability with the broader literature. The biomarker analysis, while based on paired pre–post measurements, is restricted to the 1–3 month subgroup and lacks a concurrent comparator group, limiting interpretation of the observed changes in the absence of a reference for background biological variation.

Concerning the absence of adjusted analyses, the absence of a concurrent control or non-exposure comparator group, combined with the lack of individual baseline symptom measurements, represents a fundamental analytic constraint. Observed associations cannot be separated from background variation, placebo effects, regression to the mean, or concurrent lifestyle and behavioral changes. Individual-level covariate data—including BMI, menopausal status, comorbidity burden, and concurrent medications or supplementation—were not available in the dataset and therefore adjusted regression modeling could not be performed. As a result, confounding by indication cannot be statistically controlled, and the observed associations should be interpreted with corresponding caution.

Several additional limitation merit mention. Delivery modality (topical, oral, or subcutaneous) was not captured in the survey instrument, precluding any assessment of route-specific effects. The racial, ethnic, and socioeconomic composition of the cohort was not recorded, limiting generalizability to more diverse populations. Adverse effects and side effect profiles associated with TRT were not systematically captured in the survey instrument and therefore cannot be reported. Additionally, individual-level linkage between biomarker changes and symptom outcomes was not available in the dataset, precluding correlation analyses between hormonal changes and clinical improvements. Such analyses would be valuable in establishing mechanistic relationships and should be prioritized in future prospectively designed studies. This is a meaningful clinical omission given the known androgenic side effects of testosterone therapy in women, including acne, hirsutism, and voice changes, and future studies should incorporate structured adverse event monitoring. The telehealth-based recruitment model introduces additional constraints on generalizability. Participants self-selected into a commercially available hormone optimization platform, which likely enriches the cohort with individuals who are health-engaged, digitally literate, and motivated to pursue proactive hormonal management; characteristics that may not be representative of the broader population of women experiencing testosterone insufficiency. Furthermore, the individualized, biomarker-guided dosing protocol used here differs substantially from the fixed-dose regimens employed in most published RCTs, making direct comparisons with the existing literature difficult and limiting the extent to which these findings can inform standardized clinical practice guidelines. Taken together, these limitations indicate that the present findings are strictly associative in nature. Confirmation requires larger prospective studies with validated instruments, individual baseline assessments, covariate-adjusted analyses, concurrent comparator groups, and representation of diverse populations.

## 5. Conclusions

This cross-sectional and retrospective observational study found that individualized TRT in women was associated with broad subjective improvement across eight symptom domains including energy, mood, cognition, and sexual health with a progressive duration–response relationship and favorable changes in all five biomarkers assessed at 12 weeks. These findings extend the existing evidence base beyond sexual function and suggest that the therapeutic value of testosterone in women may be considerably broader than current clinical and regulatory frameworks reflect. However, these findings are subject to important methodological limitations detailed in the Limitations section, and confirmation requires larger prospective controlled studies with validated instruments, diverse populations, and extended follow-up.

## Figures and Tables

**Table 1:** TRT, testosterone replacement therapy; SD, standard deviation. Participants received testosterone via topical (daily application), oral (troche/ODT/RDT; once or twice daily), or subcutaneous injection (once or twice weekly) routes. Dosing was individualized to baseline serum hormone levels, symptom profile, and clinical response, with titration targeting serum total testosterone concentrations of 25–50 ng/dL. All participants were managed by a licensed provider, with dose adjustments guided by clinical response, laboratory results, and side effect profile at regular intervals. Delivery modality distribution was not captured in the survey instrument.

Characteristic	Value
Age (years)	
Mean ± SD	45.7 ± 7.1
Median (range)	45 (27–78)
Age Distribution, n (%)	
<40 years	50 (16.6)
40–49 years	166 (50.0)
50–59 years	88 (26.5)
≥60 years	23 (6.9)
TRT Duration, n (%)	
1 month	37 (11.1)
1–3 months	112 (33.7)
4–6 months	80 (24.1)
7–12 months	70 (21.1)
>12 months	33 (9.9)

Patient Demographics and Clinical Characteristics (n = 332).

**Table 2:** SD, standard deviation; CI, confidence interval; SHBG, sex hormone-binding globulin. All changes are in the expected physiological direction. Cohen’s d reported as absolute values. Favorable direction of change: total testosterone ↑, free testosterone ↑, hemoglobin ↑; SHBG ↓, triglycerides ↓.

Marker	Baseline Mean ± SD	3-mo Mean ± SD	Mean Change	% Change	95% CI (Change)	Cohen’s d
Total Testosterone (ng/dL)	16.8 ± 4.4	42.3 ± 8.7	+25.52	+151.80%	(24.25, 26.79)	3.60
Free Testosterone (pg/mL)	1.2 ± 0.3	3.8 ± 0.9	+2.55	+216.70%	(2.40, 2.70)	3.01
SHBG (nmol/L)	74.4 ± 15.5	64.5 ± 15.9	−10.94	−13.30%	(−11.6, −8.81)	1.57
Hemoglobin (g/dL)	12.7 ± 0.6	13.4 ± 0.7	+0.67	+5.50%	(0.61, 0.73)	2.03
Triglycerides (mg/dL)	159.6 ± 30.5	139.5 ± 34.5	−20.4	−12.60%	(−22.84, −17.25)	1.28

Paired Biomarker Changes, Baseline vs. 3-mo TRT (n = 120).

## Data Availability

The original contributions presented in this study are included in the article. Further inquiries can be directed to the corresponding author.
